# External control arms for rare diseases: building a body of supporting evidence

**DOI:** 10.1007/s10928-023-09858-8

**Published:** 2023-04-24

**Authors:** Artak Khachatryan, Stephanie H Read, Terri Madison

**Affiliations:** 1 Evidence and Access, Certara, UK; 2 Evidence and Access, Certara, USA

**Keywords:** Real-world data, Real-world evidence, External control, Historical control, Rare disease

## Abstract

Comparator arms in randomized clinical trials may be impractical and/or unethical to assemble in rare diseases. In the absence of comparator arms, evidence generated from external control studies has been used to support successful regulatory submissions and health technology assessments (HTA). However, conducting robust and rigorous external control arm studies is challenging and despite all efforts, residual biases may remain. As a result, regulatory and HTA agencies may request additional external control analyses so that decisions may be made based upon a body of supporting evidence.

This paper introduces external control studies and provides an overview of the key methodological issues to be considered in the design of these studies. A series of case studies are presented in which evidence derived from one or more external controls was submitted to regulatory and HTA agencies to provide support for the consistency of findings.

## Introduction

Randomized clinical trials (RCTs) remain the most reliable study design for identifying whether differences in study outcomes may be attributed to the intervention. By promoting balance in measured and unmeasured prognostic characteristics between treatment and control arms, RCTs are less susceptible to bias and spurious causality than observational studies. Despite the obvious advantages, conducting RCTs in rare diseases may be unfeasible or unethical. For example, assembling control groups for trials in rare, life-threatening diseases with no credible or approved standard of care treatment may be unethical. Patient populations are also likely to be less willing to participate or remain in placebo-controlled trials given the potential for being assigned to the placebo arm. This issue is particularly serious in rare conditions where the pool of potential participants is likely to be small and geographically dispersed [1].

Evidence suggests that up to 30% of clinical trials in rare diseases are prematurely discontinued primarily due to patient accrual issues, while many others do not achieve target recruitment numbers or are severely delayed by slow recruitment [2, 3]. Furthermore, those trials that do not meet recruitment targets are likely to have insufficient sample sizes to detect statistically significant differences. To overcome issues relating to the assembly and retention of sufficiently large control arms, researchers may opt to conduct single-arm trials and supplement findings with data from external control arms.

According to the International Council on Harmonization of Technical Requirements for Registration of Pharmaceuticals for Human use, E10 guidelines, studies which utilize external control arms are defined as *“one in which the control group consists of patients who are not part of the same randomized study as the group receiving the investigational agent i.e., there is no concurrently randomized control group”* [4]. Evidence from external controls can serve to support regulatory decision making by supplementing data of treatment efficacy and safety from single-arm trials. There is also the potential to use external controls to support the label expansion of approved therapies and to evaluate real-world safety of interventions by contributing contextual external control data. External control arms can be derived from previous randomized control trials as well as from real-world data such as electronic healthcare records, disease registries, claims databases, or chart review data. There are two major categories of external controls: contemporaneous (or concurrent) external controls and historical (or non-concurrent) controls. Concurrent external controls are composed of a group of people from the same time period but from another setting while historical controls are composed of a group of people from an earlier time period.

Regulatory agencies including the United States (US) Food and Drug Administration (FDA) and European Medicines Agency (EMA), as well as Health Technology Assessment (HTA) agencies, such as the Canadian Agency for Drugs and Technologies in Health (CADTH), recognize the need for flexibility in the definition of control populations. However, externally controlled trial designs require case-by-case assessment and are likely to be more acceptable to regulators and HTA agencies in disease areas with high unmet need, with poor prognosis, with large effect sizes or indisputable primary outcomes (e.g., mortality), and/or in which it would be impractical or impossible to conduct RCTs due to ethical concerns or required sample sizes.

Despite the rising number of marketing authorization applications to EMA and FDA with single arm pivotal studies over the last decade [5–8], external control arm studies are challenging to conduct and even with the application of robust methodology, may be subject to bias. Accordingly, in a recently published draft guidance for industry the FDA stated that *“in many situations, however, the likelihood of credibly demonstrating the effectiveness of a drug of interest with an external control is low*” [9]. In light of this statement and in cases where RCTs are unethical or unfeasible, it may be appropriate to present a body of evidence from various sources of external controls, including advanced analyses that use summary-level estimates obtained by pooling information from similar patient populations in the literature, to alleviate concerns regarding bias and to provide evidence of consistency of findings.

The aim of this paper is to describe some of the methodological challenges associated with conducting external control arm studies and to present a series of case studies in which a body of evidence from external control studies were submitted to regulatory agencies to confirm the consistency of findings.

## Methodological considerations

Conducting external control studies is methodologically challenging and there are many examples of evidence generated using external controls being rejected by regulatory agencies and HTA agencies due to concerns relating to confounding and bias [10]. Before embarking on a study using external control data, it is essential to conduct a comprehensive feasibility assessment to establish the suitability of available data sources to act as external control arms, to consider the availability of objective and indisputable outcomes, and to consult with the relevant regulatory agency to confirm the appropriateness of the proposed approach.

Where external controls are derived from real-world data sources, the initial feasibility assessment should confirm several important features of the data source. First, it should confirm that the database holds data for a target population that is largely comparable to the trial participants. Without randomization, a key source of bias in externally controlled trials is systematic differences in patient characteristics known to affect the risk of the endpoint, such as age, disease duration, and severity. Differences in these attributes mean that disparities in the rate or value of observed endpoints between the trial and external control arm cannot be ascribed to the intervention. In settings where external controls are to be directly compared to patients from single-arm trials, it is especially important that the trial eligibility criteria are largely operational in the real-world data source to attenuate the risk of this bias. In such scenarios, it is necessary to confirm whether patient management practices, patient characteristics, and disease diagnosis are consistent across the trial setting and the chosen data source. This may necessitate that the external control arm comprises patients from the same or similar geographic region and with a similar enrolment timeframe to account for differences in standard of care, access to care, and patient case-mix. During the feasibility assessment it is also necessary to confirm that the data source captures data on key confounders so that the comparability of the groups may be assessed and that appropriate analytic approaches such as propensity score methods can be implemented. Where the aim of an external control study is to establish benchmark and contextual data, the requirement that the external control arm closely matches the trial arm may be more relaxed.

Second, the feasibility assessment should confirm that the data source additionally captures accurate and valid data on treatments and endpoints. The availability of these data elements is likely to vary according to the type of data source. For example, claims databases are likely to have less data relating to patient clinical outcomes such as progression as compared to electronic healthcare records and disease registries. Data relating to potential comparator or concomitant treatments, including timing, frequency, dose, and duration is important to confirm the appropriateness of the comparator group and the comparability of the groups. While in clinical trials there may be specific rules in place when a trial participant is administered other non-investigational drugs during follow-up, no such rules will have been in place in real-world settings. Being able to account for these additional differences between groups is another important consideration when selecting the data source and specifying the study population for the external control arm. A key challenge in the development of external arms is the alignment of endpoint definitions. In trials, the method of and timing of endpoint assessment is carefully defined per protocol; a feature that is unlikely replicable in most real-world data sources. Many endpoints in real-world data are likely to be captured using different criteria, more sparingly, and their assessment is likely to be influenced by suspected clinical need. As such, the patients with measured endpoints may reflect a more unwell patient population than those patients without measured endpoints. Endpoint assessments in external control arms derived from real-world data are also not typically blinded, which may be an additional source of bias.

Once an appropriate data source is chosen, careful consideration regarding the study design is needed. In particular, alignment of index dates across trial and external control arms is essential to avoid immortal time biases. Similarly, robust analytic approaches to address confounding such as individual matching and propensity score approaches should be implemented. However, even with the application of these methods, the potential for unmeasured confounding remains a key concern of studies using external controls.

Given the complexity of assembling a reasonable external control arm, in some circumstances it may be necessary to submit more than one analysis using different types of external controls to substantiate evidence of treatment efficacy and/or safety. This was illustrated in the approvals of blinatumomab and avelumab.

## Blinatumomab case study

Blinatumomab is a drug to treat Philadelphia chromosome-negative relapsed or refractory precursor B-cell acute lymphoblastic leukemia, a rare and aggressive cancer. The initial indication was granted accelerated approval by the FDA in 2014 and EMA in 2015 based upon findings from a single-arm, open-label phase 2 trial (BLAST) and supplemental data derived from a historical control arm. [11] A second external control arm study using summary-level outcome estimates from previous clinical trials provided further support for the approval of the drug. Based upon confirmatory findings from a phase 3 trial, blinatumomab was granted full approval from the FDA and EMA in 2017 and 2018, respectively [12].

The primary objective of the BLAST trial was to demonstrate that the rate of complete remission (CR) or complete remission with partial hematological recovery of peripheral blood counts (CRh*) exceeded a pre-specified efficacy threshold of 30%. The trial included 185 eligible patients and demonstrated a rate of CR + CRh* of 42% (95% confidence interval: 34-49%). To provide contextual data to results, specifically relating to the acceptability of the 30% efficacy threshold, a historical control arm study was performed. The historical control arm comprised data for 694 patients receiving existing salvage therapies from 13 US and European study groups and treatment centers [13]. Patients for the historical control arm were selected based on key eligibility criteria of the BLAST trial. The proportion of patients with CR was estimated using two separate analytic approaches: (1) weighting analyses with weighting by the frequency distribution of prognostic factors in the BLAST trial and (2) inverse propensity weighting methods following the merging of data from the BLAST trial and historical control arm. The weighted analysis demonstrated an observed CR rate of 24% (95% CI: 20-27%) therefore providing reassurance of the appropriateness of the pre-specified 30% efficacy threshold.

To provide further support of the efficacy of blinatumomab relative to existing therapies, findings from a model-based meta-analysis study (‘synthetic control arm’) were presented. Data from 21 clinical studies published between 1995 and 2012 were extracted using Certara’s Clinical Outcomes Database Explorer (CODEx) Clinical Trial Outcomes Databases and used to developed mixed-effects meta-analysis models [14]. These models were subsequently used to simulate the effect of blinatumomab relative to existing salvage therapies. The estimated CR rate of existing therapies was 13% (95% CI 4%-34%) and the odds ratio of CR for blinatumomab compared to existing therapies was 3.50 (95% CI: 1.63–8.40) [15].

With the submission of these findings, an FDA statistical reviewer commented *“Although retrospective historical studies may not be directly comparable to prospective clinical trials, each of the historical studies provided was conducted in a large number of patients; accounted for differences in patient characteristics between studies; and independently derived a CR rate not exceeding 30% for patients receiving salvage therapies”* [16].

Subsequently, as evidenced in blinatumomab case study, defined and comparable endpoints, appropriate analytical techniques, demonstration of marked observed differences, and consistency of results between various sources of external controls maximizes the chances of success for the approval of such studies.

## Avelumab case study

Avelumab is a drug approved for the treatment of metastatic Merkel cell carcinoma, a rare and progressive skin cancer with poor prognosis. It received accelerated approval by the FDA in 2017 and conditional EMA approval in 2017 [17, 18]. These approvals were based upon findings from the JAVELIN Merkel 200 single arm phase 2 trial and two historical control populations. The JAVELIN Merkel 200 trial enrolled 88 patients and demonstrated an objective response rate of 31.8% (95.9% confidence interval 21.9-43.1%). To address the lack of comparator arm and confirm the objective response rate among control patients, two historical control populations were formed. The first population was comprised of 20 patients in US oncology settings, while the second population was derived from a Merkel cell carcinoma registry comprising data for 34 patients attending academic settings in Germany, Austria, and Switzerland. The observed objective response rate for the US and European registry was 20% (95% confidence interval: 5.7-43.7%) and 8.8% (95% confidence interval: 1.9-23.7%), respectively. EMA reviewer concluded “*Taking into account the caveats with registries and observational studies, the data can only be considered as supportive as there were divergences observed in terms of objective response rates in the registry study and in published clinical experience in first line treatment*”.

Subsequently, as illustrated in the avelumab case study, whereas the use of different sources of external controls may increase uncertainty if the obtained results are not entirely consistent, they may nevertheless contribute to the body of supportive evidence, being acknowledged by regulators.

## Erliponase alfa case study

The approval of erliponase alfa provides supporting evidence of the value in conducting sensitivity analyses to demonstrate robustness of study findings. Erliponase alfa is a treatment for neuronal ceroid lipofuscinosis type 2 disease, a rare pediatric neurological disease. This drug was approved in 2017 by the FDA and EMA on the basis of comparisons between the primary outcomes of 23 treated patients participating in a phase 1/2 open-label single arm trial and 42 historical controls derived from the DEM-CHILD database, a European registry [19]. Patients were 1:1 matched according to baseline motor-language score, age, and genotype. The primary outcome was time until a 2-point decline in the score on the motor and language domains of the CLN2 Clinical Rating Scale. Compared with historical controls, treated patients were less likely to have a 2-point decline in the combined motor-language score (hazard ratio, 0.08; 95% confidence interval: 0.02–0.23). To confirm the robustness and consistency of the findings, sensitivity analyses were performed in which 1:many matching was undertaken and in which the absence of unreversed 1-point decline was the endpoint. The value in conducting additional sensitivity analyses was highlighted in reviews undertaken by the EMA: “*important evidence of efficacy comes from comparisons with a longitudinal untreated historical control group. The applicant has applied acceptable methods (most importantly the 1:1 matching) to account for potential bias and provided several sensitivity analyses that support the robustness of the findings”.*

Again, this case study provides valuable insight into the importance of generating a body of evidence of the efficacy of the new treatment to reassure reviewers.

## Defibrotide sodium case study

Defibrotide sodium was approved by the FDA and EMA for the treatment of hepatic veno-occlusive disease following hematopoietic stem-cell transplantation. This is a rare, multi-organ dysfunction disease with an estimated mortality rate of 80% and at the time of FDA and EMA submission, with no approved therapy [20, 21]. The efficacy of defibrotide sodium was initially demonstrated in a multicenter, open-label study assessing CR at day 100 among 102 eligible patients who were administered defibrotide sodium compared to 32 external (historical) control patients who received standard of care. Compared with the historical controls, the treatment group demonstrated improved CR rates (23.5% [95% CI: 15.3–31.8%] vs. 9.4% [0.0-19.5%]). However, EMA reviewers regarded this evidence as unsatisfactory since the original external control arm contained 86 patients, but this was reduced to 32 patients following an interim analysis. The results of the analysis using the original 86 patients showed no difference in response rates between groups. As a result, the EMA did not consider the 32-patient external control group as an acceptable control and subsequently refused marketing authorization of defibrotide sodium in March 2013. Upon re-examination of the data in July 2013, including with additional analyses of an external comparator from a US patient registry, the EMA granted marketing authorization. Evidence from the US registry demonstrated that survival by day + 100 for patients treated with defibrotide sodium and standard of care was 39% compared with 31% for patients receiving standard of care only. Based upon these data, the EMA concluded that the “*weight of the evidence suggests a survival benefit for defibrotide in the treatment of severe VOD*”. The presentation of additional external control data therefore sufficiently strengthened the body of evidence to enable the approval of this treatment.

## Collaboration with relevant agencies

With an ever-increasing number of available real-world data sources and an array of methodological approaches to be considered, early discussion with regulators through scientific advice on the feasibility and acceptability of the proposed methodology and study protocol to meet regulatory, HTA needs, and the legal requirement for clinical trials is essential. As highlighted in a recently published draft guidance for industry by the FDA: *“Sponsors should consult with the relevant FDA review division early in a drug development program about whether it is reasonable to conduct an externally controlled trial instead of a randomized controlled trial. As part of these discussions, sponsors should provide a detailed description of the (1) reasons why the proposed study design is appropriate, (2) proposed data sources for the external control arm and an explanation of why they are fit for use, (3) planned statistical analyses, and (4) plans to address FDA’s expectations for the submission of data.”.* [9].

## Summary

The number of regulatory submissions and health technology assessments which present real-world data alongside trial data has risen considerably in the last decade. Nonetheless, the use of external controls is appropriate only in specific circumstances and following comprehensive feasibility assessment. Despite robust efforts to eliminate bias and unmeasured confounding, concerns are likely to persist. Accordingly, it may be necessary to present supportive and confirmatory evidence derived from studies which implement multiple analytic approaches and/or data from distinct external controls to demonstrate consistency, robustness and replicability of findings.
