# Correction: External control arms for rare diseases: building a body of supporting evidence

**DOI:** 10.1007/s10928-024-09900-3

**Published:** 2024-01-19

**Authors:** Artak Khachatryan, Stephanie H Read, Terri Madison

**Affiliations:** Evidence and Access, Certara, UK

**Correction to: Journal of Pharmacokinetics and Pharmacodynamics (2023) 50:501-506**

10.1007/s10928-023-09858-8

In this article, under the section heading ‘Blinatumomab case study’ third paragraph should have been read as below:

To provide further support of the efficacy of blinatumomab relative to existing therapies, findings from a model-based meta-analysis study (‘synthetic control arm’) were presented. Data from 21 clinical studies published between 1995 and 2012 were manually extracted and used to develop mixed-effects meta-analysis models. In hindsight, this process can be substantially streamlined when using databases such as Certara’s Clinical Outcomes Database Explorer (CODEx) Clinical Trial Outcomes Databases [1]. The blinatumomab models were subsequently used to simulate the effect of blinatumomab relative to existing salvage therapies. The estimated CR rate of existing therapies was 13% (95% CI 4%-34%) and the odds ratio of CR for blinatumomab compared to existing therapies was 3.50 (95% CI: 1.63–8.40) [2].
